# Effect of the Ratio of Soy, Pea, and Wheat Proteins on the Sensory and Textural Properties of a Minced Plant-Based Meat Analogue for Patty Applications

**DOI:** 10.3390/foods15173049

**Published:** 2026-08-28

**Authors:** Linara Murat, Daulet Aitmukhanbetov, Bakhyt Shaimenova, Amirsana Kiykbay, Dina Khamitova, Alexandr Borovskiy, Sayagul Tazhina, Gaukhar Akshorayeva, Indira Temirova, Gulnazym Ospankulova

**Affiliations:** 1 Department of Food Technology and Processing Products, Technical Faculty, Saken Seifullin Kazakh Agrotechnical University, Zhenis Avenue, 62, Astana 010000, Kazakhstan; linaraazamatkyzy@mail.ru (L.M.); daulet-kerei@mail.ru (D.A.); bshaymenova@mail.ru (B.S.); sanabayq@gmail.com (A.K.); dina.khamitova@nu.edu.kz (D.K.); a.yu.borovskiy@gmail.com (A.B.); sayagultazhina@gmail.com (S.T.); gaukharmadok@gmail.com (G.A.); indira_t85@mail.ru (I.T.); 2 Regional Technological Park QazAgroOrganic LLP, Zhenis Avenue 62b, Astana 010000, Kazakhstan; 3 Department of Biological Sciences, School of Sciences and Humanities, Nazarbayev University, Astana 010000, Kazakhstan; 4 Aron R&D Co. LLP, Akmeshit Street 5, Astana 010000, Kazakhstan

**Keywords:** PBMA, minced meat, textured soy protein, textured wheat, textured pea protein, textured vegetable protein, sensory analysis, texture profile analysis

## Abstract

The development of plant-based meat analogues with defined physicochemical, textural, techno-functional, and sensory properties is relevant amid the growing demand for sustainable protein sources. This study aimed to develop and mathematically model a plant-based meat analogue based on textured soy, pea, and wheat proteins in a minced meat form. Seven models of minced meat mixtures were prepared with the help of Mixture Design module of the Minitab program. Extreme Vertices was selected as the Type of Design and three types of textured vegetable protein were chosen as the variable components. Physicochemical indicators; techno-functional properties, including water absorption capacity, water absorption index, and oil absorption capacity; texture profile parameters; and sensory attributes were determined. Protein content ranged from 18.42 to 21.29% and caloric value from 181.11 to 184.02 kcal/100 g. All models of minced plant-based meat analogue formulations showed high techno-functional indicators. Texture profile analysis revealed differences between raw samples, whereas after cooking their textural characteristics approached those of the cooked beef control. Sensory evaluation showed that the best characteristics were obtained in formulations containing all three plant proteins, with predominance of soy and pea components. Mixture regression analysis and response surface optimization were used to evaluate protein effects and identify the optimal ratio in the Mixture Design module of the Minitab program. The composite sensory index and the hardness of the raw minced mixtures were used as the two responses. The composite sensory index was maximized while hardness was set to a target value of 4 N, corresponding to that of raw beef mince. Optimization determined the optimal textured protein mass fraction ratio: textured soy protein (0.598): textured pea protein (0.250): textured wheat gluten (0.152). These results can support further modelling of plant-based meat analogue formulations with improved sensory and textural properties.

## 1. Introduction

Products of animal origin have traditionally formed the basis of the diet of the population of Kazakhstan. According to data from the Bureau of National Statistics, the average annual per capita consumption in 2024 was 82.6 kg for meat and meat products, 232.2 kg for milk and dairy products, and 205.8 units for eggs [1]. However, with the rising standard of living, consumers are showing increasing interest in healthy dietary patterns, the consumption of organic products, and the ethical and environmental dimensions of consumer behaviour [2]. In this regard, the development of plant-based meat analogues based on plant raw materials is one of the promising directions in the food industry.

In addition to a balanced amino acid composition and protein content, techno-functional, structural, mechanical, and textural characteristics play a role in the creation of meat analogues [3]. For minced products, the water- and fat-binding capacity, which determine product yield and losses during heat treatment, are of considerable importance, as are hardness, cohesiveness, and chewiness, which create meat-like sensations during mastication [4,5].

The most common raw material for producing TVP is soy protein, whose texturates are distinguished by a high protein content and the ability to form a fibrous structure [6]. Pea protein is used for its high digestibility and amino acid profile, although it may impart characteristic beany notes of taste and aroma [7,8,9,10,11]. Wheat gluten, which forms a viscoelastic network of gliadins and glutenins, is used as a structure-forming component of protein blends [12,13,14,15]. Texturization is carried out by extrusion: low-moisture extrusion yields dry porous texturates that require subsequent rehydration, whereas high-moisture extrusion yields products with a pronounced anisotropic structure [16].

The combination of plant proteins has been studied mainly in the context of joint extrusion. Schreuders et al. [17] showed that the composition of the protein system and the processing temperature determine the mechanical properties of binary pea– and soy–gluten systems. Lee et al. [18] examined the effect of the soy-to-pea protein ratio on the properties of high-moisture extrudates. Flory et al. [19] varied the ratio of soy, pea, and wheat proteins, defining it along the swelling axes of the raw material. Webb et al. [20] compared these proteins and the low-moisture extrudates obtained from them at the level of the textured raw material itself.

Ternary systems have also been studied using mixture design. Elshamy et al. [21]. used a mixture design to adjust the ratio of faba bean, pea, and soy proteins in high-moisture extrudates, targeting the texture of a commercial plant-based analogue. Chitra Devi et al. [22] used a mixture design to optimize the ratio of pea and soy proteins and wheat gluten with respect to hardness and acceptability in freeze-structured products. For comminuted plant-based products, mixture design has been applied to the ratio of texturate, fat, and water and to the proportions of jackfruit, textured soy protein, mushroom, and gluten [23,24].

In the present work, the optimization variable was the ratio of three texturates produced by separate low-moisture extrusion and combined during preparation of the minced mass; unlike the studies discussed above, the proteins were not structured jointly, and the ratio was defined at the stage of the finished mince. The way the textural response was defined also differs from established approaches: the hardness of the raw mince was not maximized but was brought to the value measured for the beef control. This keeps hardness within the range characteristic of meat and allows the formulation to be optimized simultaneously with respect to hardness and the composite sensory index.

The aim of the present work was to determine the ratio of soy, pea, and wheat texturates produced by separate low-moisture extrusion that provides the highest composite sensory index of a comminuted plant-based meat analogue at a raw-mince hardness matching that measured for beef.

## 2. Materials and Methods

### 2.1. Materials and Reagents

Commercial wheat protein isolate (BioOperations LLP, Tayinsha, Kazakhstan) with a protein content declared by the manufacturer of 70 g per 100 g of product was used as the protein raw material for extrusion texturization. The soybeans and peas used to obtain the protein isolates were purchased from the retail network (Astana, Kazakhstan) and were stored at 18 °C until the start of the experiment. Minced beef, used as the control sample, was purchased from a local commercial store in Astana, Kazakhstan. Vegetables (onion, carrot, beet) were purchased from local suppliers in Astana, Kazakhstan. Commercial wheat starch was supplied by LLP “BioOperations” (Tayinsha, Kazakhstan). Sunflower vegetable oil was supplied from the local markets in Astana, Kazakhstan, and produced by JSC “May” (Ust-Kamenogorsk, Kazakhstan). Analytical-grade reagents were used for proximate composition analysis. Sulfuric acid (95–97%, CARLO ERBA) and hydrochloric acid (37%, CARLO ERBA) were purchased from LLP “BioHimPribor” (Almaty, Kazakhstan). n-Hexane (95%, Atom Scientific Ltd., Hyde, United Kingdom), petroleum ether (Honeywell/Riedel-de Haën), and anhydrous sodium sulfate (99+%, Thermo Fisher Scientific, Waltham, MA, USA) were purchased from LLP “AlHimik” (Almaty, Kazakhstan).

### 2.2. Preparation and Analysis of Protein Isolates

Soy and pea protein isolates were obtained by alkaline extraction followed by isoelectric precipitation according to the method of Narale et al. [5] with modifications. The beans were milled into flour and defatted with n-hexane in a Soxhlet apparatus. The defatted flour was suspended in distilled water at a ratio of 1:10 (*w*/*v*), the pH was adjusted to 9.0 with 0.1 M NaOH solution, and the mixture was stirred for 1 h at 30 °C. The suspension was centrifuged (5000 rpm, 20 min, 4 °C), and the supernatant was collected. The protein was precipitated by adjusting the pH of the supernatant to 4.5 with hydrochloric acid solution. The precipitate was separated by centrifugation under the same conditions, washed with distilled water, freeze-dried in a HyperCOOL 9090 Plus unit (Hanil Scientific Inc., Gimpo, Republic of Korea), and stored at 4 °C. The protein recovery was 85.21 ± 0.09% for soy and 83.25 ± 0.15% for pea.

The proximate chemical composition of the soy, pea, and wheat isolates was determined by the methods presented in Section 2.5; the results are given in Section 3.1.

The solubility of wheat, soy, and pea proteins was determined according to the method of Narale et al. [5], with minor modifications. The isolates were dispersed in distilled water to a concentration of 1 mg/mL, and the pH was adjusted to 7.0. After vortex mixing for 20 min, the samples were centrifuged at 5000 rpm for 10 min (a refrigerated centrifuge Frontier FC5816R, OHAUS Corporation, Parsippany, NJ, USA). The protein content in the supernatant was determined by the biuret method. Protein solubility was expressed as a percentage of the ratio of soluble protein content to the total protein content in the sample. The protein solubility was calculated according to the following equation:

(1) $$Protein\;solubility=\frac{Protein\;content\;of\;supernatant}{total\;protein\;content}\times 100.$$

### 2.3. Extrusion Texturization of Plant Protein

Each protein isolate was texturized separately by low-moisture extrusion according to the previously described method [4]. The three isolates were processed consecutively on the same equipment under identical settings, with flushing and purging of the barrel between loads to preclude cross-contamination. For each isolate, a single extrusion run was performed, and the resulting texturates were used for all model formulations. The isolates were not blended prior to extrusion; the ratio of the textured proteins was set at the stage of preparation of the minced mixtures (Section 2.4).

Extrusion was performed on a laboratory extruder PE-20 (LLP “AGROTEKHSERVIS-12”, Kostanay, Kazakhstan) with a throughput of 20 kg/h, driven by a three-phase electric motor with a rated power of 4 kW. Each isolate was extruded at a feed moisture content of 30% (*w*/*w*) and a barrel temperature of 100 °C. The resulting protein texturates were dried at 60 °C for 2–3 h, after which they were ground on a roller mill to obtain a 2–5 mm fraction and stored until further use at room temperature.

### 2.4. Preparation of Plant-Based Meat Analogue Samples

Samples of the plant-based meat analogue (PBMA) were prepared on the basis of the procedure described previously, with some modifications [19]. As the protein base, textured soy, pea, and wheat proteins obtained beforehand after low-moisture extrusion and grinding were used.

Freeze-dried powders of onion, carrot, and table beetroot were obtained using the freeze-drying method, which is widely applied in the production of vegetable powders owing to the preservation of structure, colour, and thermolabile biologically active compounds [25]. Fresh vegetables were washed, peeled, and separately cut into slices 3–5 mm thick. The prepared raw material was evenly distributed in a single layer on trays and pre-frozen at −20 °C for 12 h.

Freeze-drying was performed in a HyperCOOL 9090 Plus unit (Hanil Scientific Inc., Gimpo, Republic of Korea) with a condenser temperature of −90 °C until constant weight was reached. The resulting freeze-dried vegetables were ground in a laboratory mill to a powdered state and sieved through a sieve with a mesh size of 0.5 mm. The finished powders were placed in airtight light-proof containers and stored at 4 ± 1 °C until use in the preparation of the plant-based patties. The selected freeze-drying conditions are consistent with previously published studies on the production of freeze-dried vegetable powders and beetroot [25].

Seven model PBMA formulations were prepared, differing in the ratio of textured soy, pea, and wheat proteins at a constant content of the remaining components (Table 1). Minced beef without plant-based ingredients was used as the control, to which table salt was added at the same level as in the plant-based formulations.

Model formulations of PBMA minced mixtures were prepared on the basis of blends of plant protein texturates according to Flory et al. [19], with minor modifications, and are presented in Table 1. Each patty was prepared from 100 g of the formulated mixture and formed into cylindrical patties with a diameter of 80 mm and a thickness of 20 mm.

The ground textured proteins were mixed with starch, freeze-dried vegetable powders, yeast extract, and vegetable oil according to the developed formulations. Cooled water (4–5 °C) was added to the mixture, which was then blended at 60 rpm for 20 min to achieve uniform component distribution and a homogeneous minced meat-like consistency.

After mixing, the resulting mixture was held at 4–6 °C for 3–4 h for hydration of the dry components and stabilization of the structure. The prepared PBMA samples were frozen at −20 °C and stored until further physicochemical and textural analyses. Before the physico-chemical and sensory analyses, the formed samples were subjected to heat treatment by pan-frying in a non-stick-coated pan at 150 °C for 10 min. During frying, the samples were periodically turned over to ensure uniform heating. The criterion for completion of the heat treatment was reaching a temperature of 75 °C at the geometric centre of the product, which was monitored with a needle thermometer.

### 2.5. Proximate Composition Analysis

The study of the basic chemical composition included determination of moisture, protein content, fat, total carbohydrate content, and ash according to the standard AOAC (2019) methods [26].

Moisture content was determined gravimetrically by drying samples in an oven at 105 °C to constant mass. Crude ash content was determined by incineration in a muffle furnace at 550 °C. The crude protein content was determined by the Kjeldahl method (N × 6.25) and the crude fat content by Soxhlet extraction using petroleum ether (TURBOTHERM, VACUSOG and SOXTHERM systems, C. Gerhardt GmbH & Co. KG, Königswinter, Germany). The energy content was calculated using modified Atwater conversion factors for carbohydrates, and the values were expressed in kilocalories (kcal) [26]. The total carbohydrate content was calculated by following equation:

100 − [moisture + crude protein + crude fat + crude ash](2)

The indicator calculated in this way corresponds to the total carbohydrate content and includes dietary fibre.

pH was determined using a desktop digital pH meter (S220-kit, METTLER TOLEDO, Columbus, OH, USA) after homogenising the samples with distilled water at a ratio of 1:10 (*w*/*v*).

### 2.6. Colour Characteristics

The colour of each sample was determined using a YS6010 spectrophotometer (3nh, Guangzhou, China) in the CIE L*, a*, and b* system with a 10° observer and a D65 light source. The results were presented as the mean value of measurements performed at three randomly selected points on the sample surface. The total colour difference (ΔE*) was calculated using the following equation. The ΔE* index was used to evaluate visually discernible colour changes [27]:

(3) $${\Delta E}^{*}={\left[{\left({\Delta L}^{*}\right)}^{2}+{\left({\Delta a}^{*}\right)}^{2}+{\left({\Delta b}^{*}\right)}^{2}\right]}^{1/2}$$

where ΔL*, Δa*, and Δb* are the differences between the colour coordinates of the test sample and those of the control minced beef sample after heat treatment. The spectrophotometer was calibrated against a reference white plate (L* = 98.84; a* = 0.02; b* = 0.59) before each series of measurements.

Whiteness is an important indicator of the commercial quality of a product and is determined by the combination of high lightness and low intensity of the yellow hue [28]. The whiteness index was calculated using the following formula:

(4) $$Whiteness={100-\left[{\left({100-L}^{*}\right)}^{2}a^{*2}b^{*2}\right]}^{1/2}$$

### 2.7. Techno-Functional Properties

The techno-functional properties of the PBMA in the form of minced meat samples were evaluated by the previously described method, with minor modifications [29]. Water absorption capacity (WAC) was determined by mixing 5.0 g of sample with 25 mL of distilled water in a pre-weighed centrifuge tube. Then, the obtained mixture was stirred for 5 min followed by centrifugation at 3000 rpm for 15 min (a refrigerated centrifuge Frontier FC5816R, OHAUS Corporation, Parsippany, NJ, USA). After centrifugation, the supernatant was removed, and the hydrated pellet was weighed.

To determine the water absorption index (WAI), 1.75 g of sample was combined with 15 mL of distilled water in a pre-weighed centrifuge tube. The mixture was then heated at 70 °C for 30 min in a water bath, then centrifuged at 5000 rpm for 20 min. The resulting pellets were weighed after the removal of supernatant.

The oil absorption capacity was measured in a similar manner: 0.75 g of sample was combined with 9 mL of vegetable oil in the same pre-weighed tube. After mixing, it was centrifuged under the same conditions of 5000 rpm for 20 min. The excess oil was carefully decanted, the pellet with the retained oil was weighed, and the OAC was expressed in grams of oil per gram of sample.

### 2.8. Texture Profile Analysis

Texture profile analysis of the PBMA samples (in the raw and cooked states) was performed using the standard double-compression texture profile analysis (TPA) method, which has been used in studies of plant-based analogues of meat products [30]. The analysis was performed on a “Strukturometr ST-2” equipment (LLP “Laboratoriya kachestva”, Moscow, RF). Prior to analysis, the samples were held at room temperature (20–22 °C) for 30 min. Then, the samples of the minced mixtures and patties were cut into pieces measuring 3.0 × 3.0 cm with a thickness of 2 cm.

The hardness (N) and adhesiveness (N) of the samples were also determined under the following conditions: cylindrical probe, 30 mm in diameter; test speed 0.5 mm/s, trigger force of 0.05 N, up to a final loading at a depth of 10 mm.

The cohesiveness (%), springiness (%), gumminess and chewiness (N·mm) were measured at the following conditions: cylindrical probe, 30 mm in diameter; test speed of 0.5 mm/s, trigger force of 0.05 N, strain force of 2 N.

The elasticity (h_el_/h_tot_) of the samples was determined on the basis of data on the total (h_tot_), plastic (h_pl_), and elastic (h_el_) deformation under the following conditions: cylindrical probe, 30 mm in diameter; test speed 0.5 mm/s, trigger force of 0.05 N, strain force of 5 N.

Each group of parameters was measured under its own loading regime: hardness and adhesiveness up to a penetration depth of 10 mm, cohesiveness, springiness, gumminess and chewiness at a strain force of 2 N, and elasticity at a strain force of 5 N.

### 2.9. Sensory Evaluation

Sensory evaluation of plant-based meat analogues was carried out using a quantitative descriptive sensory profiling approach, in accordance with the general principles of sensory analysis set out in ISO 13299:2016 [31]. The present study involved only adult participants, was non-invasive and non-interventional in nature, and did not entail the collection of sensitive personal or medical data. Participation in the sensory evaluation was voluntary, and informed consent was obtained from all participants prior to tasting. The sensory protocol was reviewed and approved in accordance with the internal regulations of S. Seifullin Kazakh Agrotechnical Research University.

The sensory panel comprised eight experts who were staff members of the Institute of Engineering and Food Technology at S. Seifullin Kazakh Agrotechnical Research University. The panel included four men and four women aged 25 to 55 years, all of whom had prior experience in the sensory evaluation of food products. Prior to the main evaluation, a preliminary training and orientation session was organized for the experts, during which the sensory attributes to be evaluated, their definitions, the scoring procedure, and the rules for using the 9-point scale were explained.

Sensory attributes were rated on a structured 9-point intensity scale, where 1 corresponded to the absence or extremely weak expression of the attribute and 9 to a very high expression. For raw samples, appearance, colour and aroma were assessed. For cooked samples, the appearance of the whole patty and its cross-sectional appearance were assessed on the intact patty prior to portioning. The patties were then cut into pieces of approximately 2 × 2 × 2 cm, placed in identical glass containers, coded with random three-digit numbers and held at room temperature for approximately 30 min; these portioned samples were used for the evaluation of consistency, aroma and taste. Each attribute was rated independently according to the degree of its expression.

Each expert evaluated all seven formulations once within a single session. The order of presentation was randomized for each expert and, to the extent possible, balanced across experts. The evaluation was conducted individually under uniform lighting in the absence of extraneous odours; water was provided between samples for rinsing the mouth.

The minced beef used as the control was part of the sensory evaluation. The object of modelling was the composition of textured plant proteins, whereas the control sample was employed for comparison of the instrumentally measured characteristics. Tasting was conducted blind with three-digit coding of the samples, and a beef patty is unambiguously identified by the colour of the cut surface and the aroma.

Sensory quality is characterized by eight individual scores, whereas the mixture regression model is constructed for a single response. Reducing the individual scores to a single dimensionless indicator makes it possible to use sensory quality as a response on a par with instrumentally measured hardness and to find the optimal protein ratio beyond the experimentally studied design points. The composite sensory index (CSI) was calculated using the Harrington desirability function. Accordingly, using this methodology allows individual sensory indicators to be normalized to a dimensionless scale from 0 to 1, by means of the following function:

(5) $$d_{i}\;=\;d\;\left(z_{i}\right)\;=\exp{(-e)}^{{-z}_{i}}$$

(6) $$z_{i}=\frac{x_{i}-x_{i0}\;}{x_{i1}-x_{i0}}\;,$$

where *z_i_* is the dimensionless coded value of the *i*-th sensory attribute; *x_i_* is the mean score of the *i*-th sensory attribute for a given formulation; and *x_i_*_0_ and *x_i_*_1_ are the boundaries of the satisfactory region on the original 9-point scale, corresponding to desirability values of *d_i_*_0_ = 0.37 and *d_i_*_1_ = 0.69, respectively. For each attribute, *x_i_*_0_ and *x_i_*_1_ were taken as the lowest and the highest mean score recorded among the seven model formulations so that within each attribute, the least and most favourable formulations received the boundary desirability values.

The partial desirability values d_i_ were combined into a generalized desirability function *D*, taken as the geometric mean of the partial desirability functions over all sensory attributes (*n* = 8):

(7) $$\;D\;=\sqrt[{n}]{d_{1}d_{2}}\ldots d_{n}$$

### 2.10. Design of Experiment

Mathematical modelling of the PBMA formulations in the form of minced meat mixtures was carried out using the Mixture Design module in the Minitab v. 22.2.2 program. Three types of textured vegetable protein were chosen as the variable components: textured soy protein (TSP), textured pea protein (TPP), and textured wheat gluten (TWG). A mixture experiment approach was used to construct the design, in which not the absolute amount of each component is studied but rather their ratio to one another.

The mass fraction of textured plant proteins in the formulation was 20.16% in all model samples and was not varied; only the ratio of the soy, pea, and wheat texturates within this fraction was varied. In constructing the experimental design, the protein portion was taken as unity; therefore, the proportions of the components in Table 2 and in the regression equations are expressed as percentages of the sum of the three texturates rather than of the mass of the entire minced mixture.

Extreme Vertices was selected as the Type of Design. This option was convenient for the present work since it made it possible to cover the main variants of combining the three textured proteins without an excessive increase in the number of model formulations. This was important in order to compare both the individual influence of each protein component and the effect of their combined use within the minced mixtures.

As a result of the planning, seven formulation ratios of the textured vegetable protein were obtained. The choice of these points was related to the logic of constructing a three-component mixture. The plan included formulations containing only one type of textured protein, which made it possible to assess the contribution of each protein separately. A formulation with an equal ratio of textured soy, pea, and wheat proteins was also included, reflecting a balanced combination of the three components. In addition, formulations were selected in which one of the textured proteins predominated while the other two were present in smaller amounts. This made it possible to assess how the properties of the minced mixture change when one protein component is dominant, but a multi-component composition is maintained.

Table 2 presents the mass fractions of the textured vegetable protein in the model samples of minced mixtures.

Optimization of the plant-based meat-analogue formulations was carried out using mixture regression analysis. The mass fractions of the three types of textured vegetable protein were used as predictors. Two responses of different natures were considered simultaneously: the composite sensory index (CSI), calculated as described in Section 2.9, and the hardness of the raw minced mixture measured instrumentally, as described in Section 2.8. A full quadratic Scheffé mixture model was fitted for each response.

Simultaneous optimization with respect to the two responses was performed using the Response Optimizer module of Minitab. The CSI was set to be maximized, whereas the hardness of the raw minced mixture was not maximized but was brought to a target value of 4 N. The value corresponds to the hardness of the raw minced beef control measured under identical conditions within the present study (3.83 ± 0.30 N, Section 3.5), rounded to the nearest whole number. Defining the textural response as a target value keeps the hardness of the optimized formulation within the range characteristic of raw minced beef, while sensory acceptability is maximized. The solution was obtained as the combination of the mass fractions of the three texturates providing the highest composite desirability.

### 2.11. Statistical Analysis

All experiments were conducted at least three times, and the results were expressed as the mean ± standard error of the mean. One-way analysis of variance (ANOVA) and Tukey’s test were performed on the experimental results in Minitab Statistical Software (Version 22), with the significance level set at *p* < 0.05.

## 3. Results

### 3.1. Physicochemical and Techno-Functional Properties of the Protein Isolates

The protein isolates used for extrusion texturization differed in chemical composition and in protein solubility (Table 3). These indicators characterize the raw material supplied to the texturization stage and make it possible to interpret the differences between the model formulations described in the subsequent sections.

The protein content in the pea and soy isolates was similar and did not differ significantly at 79.36 ± 0.24% and 78.36 ± 0.24%, respectively, whereas the wheat isolate contained significantly less protein (69.37 ± 0.60%), with the highest proportion of carbohydrates (20.05 ± 0.17%). In terms of carbohydrate content, all three isolates differed significantly from one another. The fat content was lowest in the soy isolate (0.12 ± 0.01%) and highest in the wheat isolate (3.33 ± 0.23%). The soy isolate was characterized by the highest moisture content (7.80 ± 0.16%), whereas the pea and wheat isolates did not differ in this indicator. In terms of ash content, the pea isolate was significantly lower than the soy and wheat isolates, which did not differ from each other. Thus, at an equal mass fraction of the isolates in the formulation, the wheat component contributed less protein and more carbohydrates to the system than the other two.

Solubility at pH 7.0 differed significantly between the isolates (*p* < 0.05) and amounted to 16.21 ± 1.39% for the pea, 11.65 ± 0.26% for the wheat, and 7.07 ± 0.40% for the soy isolate. The absolute values for all three samples were low compared with those typical of native protein preparations, which is to be expected for isolates obtained by isoelectric precipitation followed by drying: both stages are accompanied by partial unfolding of the polypeptide chains and subsequent protein aggregation, which reduces the proportion of the soluble fraction.

The lowest solubility value, recorded for the soy isolate, indicates that it entered the extrusion texturization stage in the most denatured state.

### 3.2. Physicochemical Properties of Plant-Based Minced Meat Formulations

Physicochemical composition is one of the principal quality indicators of textured vegetable proteins, since it reflects their nutritional value and the features of formation of the product matrix. The contents of moisture, protein, fat, carbohydrates and ash, together with the energy value, are presented in Table 4 and allow the differences between the developed formulations and the meat control to be assessed.

As can be seen from the data in Table 4, the greatest variability among the indicators studied was observed for the protein and fat contents. The protein content in the model PBMA formulations was in the range from 18.42 to 21.29%, whereas in the control sample, it was 18.55 ± 0.05%. The highest protein content was characteristic of TSP (21.29 ± 0.27%), which differed statistically from some of the other formulations (*p* < 0.05). These results indicate that the protein content of the product is substantially influenced by the composition of the plant protein blend, which is consistent with the results of other studies of plant-based meat analogues [29].

More noticeable differences were associated with the fat content. Despite the close values of this indicator for all plant formulations (5.99–6.20%), the control sample contained almost three times more fat, 16.63 ± 0.27% (*p* < 0.05). The reduction in fat content was accompanied by a decrease in energy value: the caloric value of the plant formulations was 181.11–184.02 kcal/100 g, whereas in the control, it was 229.28 ± 3.03 kcal/100 g. These findings are consistent with previously reported differences between meat and plant-based products [32,33].

In contrast to protein and fat, the moisture and ash indicators remained fairly stable. No statistically significant differences between the samples were found for these parameters (*p* > 0.05): the moisture of the plant formulations was within 59.87–61.09%, and the ash content was 1.96–2.14%.

The characterization carried out covered the proximate chemical composition. In addition to these indicators, the nutritional profile of meat analogues is determined by protein digestibility and its amino acid composition, as well as by micronutrients—iron, zinc, and vitamin B12—the content and bioavailability of which differ between plant and animal raw materials [34]. In this respect, combining proteins of different botanical origin offers an advantage, since the limiting amino acids of cereal and legume proteins mutually complement each other [35]. A detailed assessment of these characteristics is beyond the scope of the present work, which is devoted to the influence of the protein ratio on textural and sensory properties.

Therefore, in this case, the main effect of replacing meat raw material with plant protein components manifested itself not in a change of the moisture or mineral composition but mainly in a reduction in the fat content and energy value while maintaining a comparable protein level in a number of formulations.

### 3.3. Colour Attributes

The colour characteristics of the samples studied depended on the ratio of the plant proteins. All samples examined had positive a* and b* values, which indicates the presence of red and yellow hues in the surface colour of the finished product. The values of the colour coordinates L*, a*, b* and of the whiteness index for the control and experimental samples are presented in Table 5.

The lightness values L* of the plant-based analogues ranged from 36.38 ± 0.78 to 46.35 ± 0.24. The highest lightness was characteristic of the equal-proportion formulation S_1_/_3_P_1_/_3_W_1_/_3_ and the lowest of TPP based on textured pea protein, which in this indicator did not differ significantly from the control sample (34.46 ± 0.50).

The higher lightness values of most plant-based analogues compared with the meat control are attributable to the absence of heme pigments, primarily myoglobin and its thermally induced derivatives, which produce the darker colour of meat after cooking. Similar differences between textured plant proteins and meat products were noted by Samard et al. [36], who associated them with differences in the chemical composition and optical properties of the protein matrices. Chiang et al. [37] showed that changing the ratio of soy protein to wheat gluten affects the appearance and colour of extruded analogues as a consequence of changes in the structure of the protein network and in the light-scattering ability of the material.

The values of the a* parameter varied from 6.66 ± 1.06 to 12.74 ± 1.13 and depended significantly on the protein ratio. The highest intensity of the red hue was established for S_1_/_6_P_2_/_3_W_1_/_6_ with a predominance of pea protein and the lowest for the formulations with a high proportion of wheat gluten, S_1_/_6_P_1_/_6_W_2_/_3_ and S_1_/_3_P_1_/_3_W_1_/_3_. The control sample occupied an intermediate position (8.76 ± 0.30) and differed significantly only from S_1_/_6_P_2_/_3_W_1_/_6_.

Significant differences were revealed in the b* indicator. The most pronounced yellowness was characteristic of TSP and S_1_/_6_P_1_/_6_W_2_/_3_—21.22 ± 1.00 and 20.49 ± 0.99, respectively—which significantly exceeded the values of all other samples, including the control (11.03 ± 0.63). The elevated b* values may be associated with the formation of intermediate products of non-enzymatic browning during heat treatment. Xiao et al. [38] noted that changes in a* and b* may indirectly reflect the intensity of the Maillard reaction in meat analogues. It should be taken into account that the CIELAB parameters are not direct indicators of the extent of this reaction; therefore, the explanation given is considered a probable mechanism of colour formation.

The whiteness index varied from 32.95 ± 0.46 to 44.78 ± 0.39 and generally corresponded to the variation in L*: the maximum value was established for S_1_/_3_P_1_/_3_W_1_/_3_ and the minimum for the control sample. The TPP formulation did not differ significantly from the control in this indicator.

The total colour difference relative to the beef control ranged from 3.74 ± 0.45 to 14.30 ± 1.05. According to the generally accepted scale of colour difference perception, ΔE values in the range from 2 to 3.5 correspond to a difference noticeable to an inexperienced observer, whereas values above 5 are perceived as different colours. The lowest values were recorded for the formulations containing pea protein—TPP (3.74 ± 0.45), S_2_/_3_P_1_/_6_W_1_/_6_ (5.01 ± 0.51), and S_1_/_6_P_2_/_3_W_1_/_6_ (6.74 ± 0.55)—which did not differ significantly from one another. The highest values were obtained for the formulations with a high proportion of wheat gluten and for the pure soy texturate—from 12.04 ± 0.79 to 14.30 ± 1.05.

Overall, the colour of the finished products was determined by the natural colour of the protein raw material, the ratio of the components, and the occurrence of non-enzymatic browning reactions during heat treatment.

### 3.4. Techno-Functional Properties of Plant Protein Mince Formulations

Techno-functional properties characterize the ability of minced systems to bind and absorb water, as well as to hold the lipid phase, which is important for their stability during forming and subsequent processing. For plant-based meat analogues, these indicators are especially important, since the distribution and retention of moisture and fat are associated with the formation of juiciness, consistency, and texture of the finished product [39]. In the present work, the water absorption index (WAI), water absorption capacity (WAC), and oil absorption capacity (OAC) were determined; the results are presented in Table 6.

The WAI values of the model PBMA formulations were in the range 1.54 ± 0.19–2.53 ± 0.42 g/g, whereas for the beef-mince control sample, this indicator was 0.89 ± 0.04 g/g. Despite the numerically higher WAI values of the plant systems, no statistically significant differences between the samples were established (*p* > 0.05). The maximum numerical WAI value was recorded for TSP, in which textured soy protein was the sole protein component. The more pronounced water absorption of this sample may be associated with the hydration properties of the soy protein ingredient, since the WAI of plant proteins and protein blends is determined by the nature of the protein raw material, its composition, and its ability to swell in an aqueous medium [40,41].

The water absorption capacity (WAC) of the model PBMA formulations varied from 0.90 ± 0.07 to 1.27 ± 0.02 g/g, whereas for the beef-mince control sample, this indicator was 0.90 ± 0.03 g/g. The maximum WAC value was recorded for TSP, in which textured soy protein was the sole protein component. This formulation significantly exceeded TPP and the control (*p* < 0.05) but did not differ from the other plant compositions. The higher WAC of TSP may be associated with the hydration properties of the soy protein ingredient. A similar relationship was described previously where the WAC of plant proteins is determined by the protein source, the structural features of the ingredient, and its ability to interact with water [4].

In terms of oil absorption capacity (OAC), all model PBMA formulations significantly exceeded the beef-mince control (*p* < 0.05). The OAC values of the plant compositions were in the range 1.76 ± 0.15 to 2.08 ± 0.26 g/g, whereas for the control sample, this indicator was 0.98 ± 0.06 g/g. At the same time, no statistically significant differences between the model PBMA formulations were found, which indicates a comparable ability of the compositions studied to hold the lipid phase. The higher OAC values of the model PBMA formulations may be associated with the features of the protein-carbohydrate structure and the physical characteristics of the plant ingredients. Webb et al. [20] also noted that the OAC of plant protein systems is determined not only by the affinity of the protein for oil but also by particle size, agglomeration, and interparticle spaces that promote oil retention.

Overall, the plant minced compositions differed from the beef-mince control sample in a higher ability to hold the lipid phase, without significant differences among themselves. In terms of hydration indicators, the highest numerical WAI and WAC values were observed for TSP. At the same time, the differences in WAI were not statistically significant, and in terms of WAC, TSP exceeded only TPP and the control. Thus, TSP can be regarded as a promising ingredient for enhancing the hydration properties of plant minced systems, whereas the oil-holding capacity was comparable for all plant compositions.

### 3.5. Texture Profile Analysis of the Plant-Based Minced Meat Formulations

Texture profile analysis of the plant-based meat analogue showed significant differences between the model formulations of minced mixtures both in the raw state and after cooking in the form of fried patties.

The highest textural indicators were characteristic of the TSP minced mixture based on textured soy protein: hardness of 7.89 N, springiness of 0.83, gumminess of 6.57, chewiness of 5.34, and elasticity of 36.37%. This may be associated with the high gel-forming ability of soy proteins. Batista et al. [42] established that soy protein isolate forms a more pronounced gel structure compared with pea and lupin isolates, which the authors attribute to a greater degree of molecular unfolding upon heating and the subsequent formation of a stronger protein network. Consequently, the predominance of textured soy protein in the TSP formulation could have promoted the formation of a denser and more elastic PBMA structure, which explains the elevated values of the TPA indicators.

The predominance of the textured wheat protein fraction in the minced mixtures S_1/6_P_1/6_W_2/3_ (66.7%) and TWG (100%) led, on the contrary, to a decrease in these indicators: hardness of 1.96 N and 0.96 N, cohesiveness of 0.34 and 0.38, gumminess of 0.66 and 0.37, chewiness of 0.3 and 0.3, and elasticity of 5.57% and 1.75%; this is consistent with previous studies [40,43,44].

The minced mixtures based on textured pea protein (TPP), as well as the mixtures with various ratios of textured vegetable proteins with a minimal content of wheat protein S_1/3_P_1/3_W_1/3_, S_2/3_P_1/6_W_1/6_, and S_1/6_P_2/3_W_1/6_, occupied an intermediate position in terms of hardness, gumminess, and chewiness. For the adhesiveness indicator, the inverse picture was observed among the minced mixtures: the lowest adhesiveness was in the TSP mixture based on textured soy protein of (−0.04) N, and the highest adhesiveness was in the mixtures based on textured pea protein TPP and S_1/6_P_2/3_W_1/6_ (−0.51) N and (−0.49) N. The remaining mixtures occupied an intermediate position. The texture profile analysis results are presented in Table 7.

Thermal treatment of the model minced mixtures in the form of fried patties substantially changed the textural profile of the finished products. After cooking, an increase in hardness, gumminess, chewiness, and elasticity was noted, which indicates the formation of a denser and more stable product structure. At the same time, adhesiveness decreased to minimal values. This is probably associated with the thermal denaturation and aggregation of plant proteins, as a result of which intermolecular interactions are enhanced and a more cohesive protein matrix is formed. Such a structure reduces the surface stickiness of the sample and decreases its adhesion to the compressing element of the instrument during TPA [45,46]. In addition, after cooking, a levelling-off of the cohesiveness (0.84–0.93) and springiness (0.79–0.92) indicators was observed among the plant-based formulations.

Thermal treatment significantly affected the TPA indicators depending on the type of textured protein predominant in the composition. Thus, the hardness, chewiness, gumminess, and elasticity of mixtures S_1/6_P_1/6_W_2/3_ and TWG based on textured wheat protein increased significantly after thermal treatment compared with the other model formulations. In contrast, the relative increase in these indicators for TSP was the smallest among the formulations, its structure having been largely developed already at the extrusion stage. The patties prepared from the minced mixture based on textured pea protein (TPP) exhibited the lowest values of hardness (14.07 N), gumminess (12.06), and chewiness (9.69).

Comparison with the beef control showed that the convergence of the textural characteristics after heat treatment depended on the particular indicator. In terms of hardness, none of the formulations differed significantly from the control. In terms of gumminess and chewiness, five of the seven formulations were comparable to the control, and only S_1_/_3_P_1_/_3_W_1_/_3_ and S_1_/_6_P_1_/_6_W_2_/_3_ exceeded it. The cohesiveness of all plant-based formulations (0.84–0.93) significantly exceeded that of the control (0.77 ± 0.02), which may reflect the formation of a more continuous protein matrix upon heating in the absence of a myofibrillar structure. Thus, the model formulations approached traditional beef patties in the indicators, characterizing the resistance of the product to compression and mastication, while retaining a more cohesive structure.

### 3.6. Analysis of Sensory Properties

**Figure 1:**
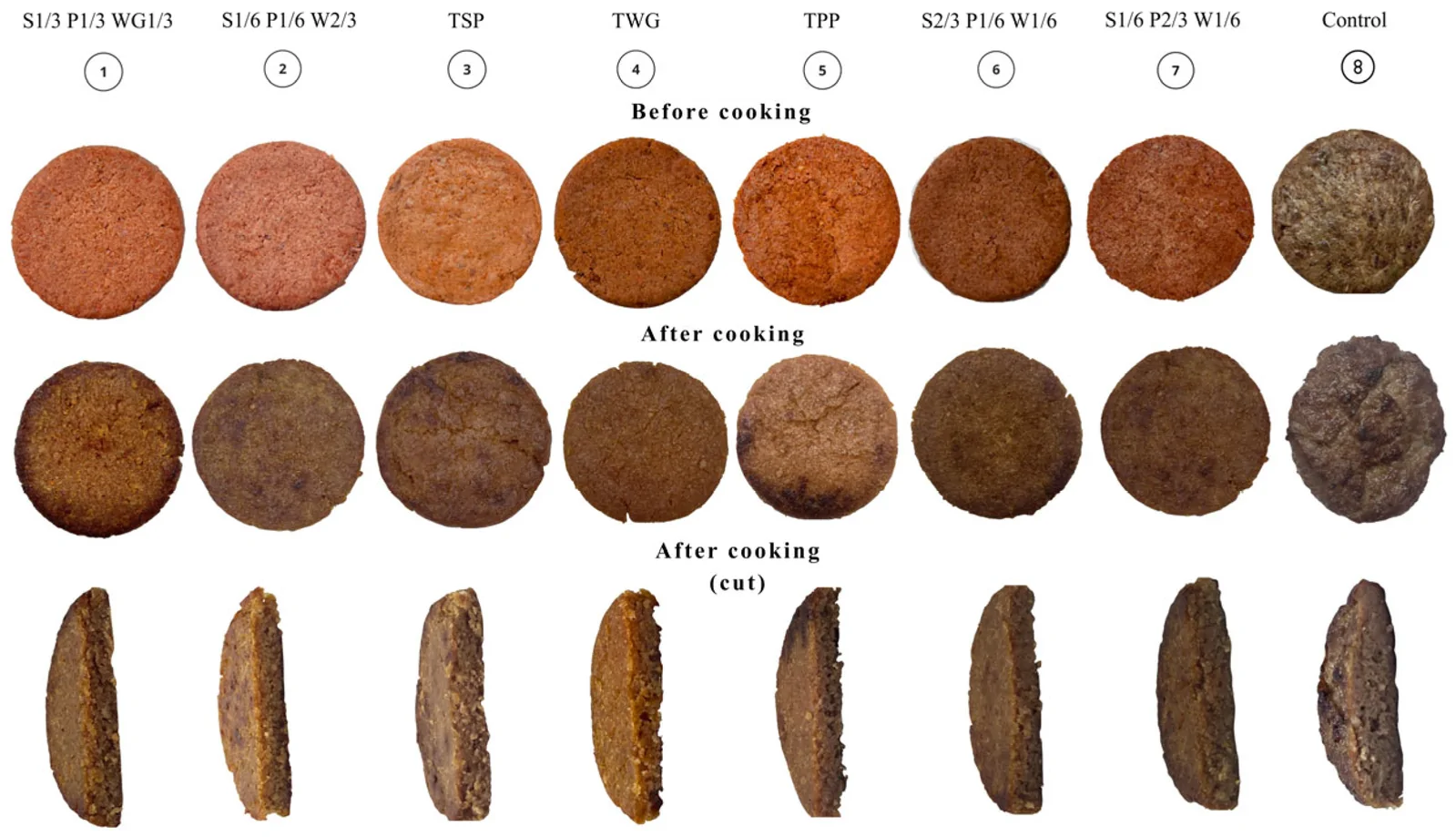
Visual characteristics of plant-based meat analogue formulations in raw patty form and after cooking in cross-section.

In assessing the quality of the finished meat analogue (Figure 1), an expert evaluation of the sensory properties of the minced mixtures in both raw form and after cooking as patties was used as a separate indicator. The expert evaluation was carried out by a panel of eight experts on a 9-point scale, and results are presented in Table 8.

**Figure 2:**
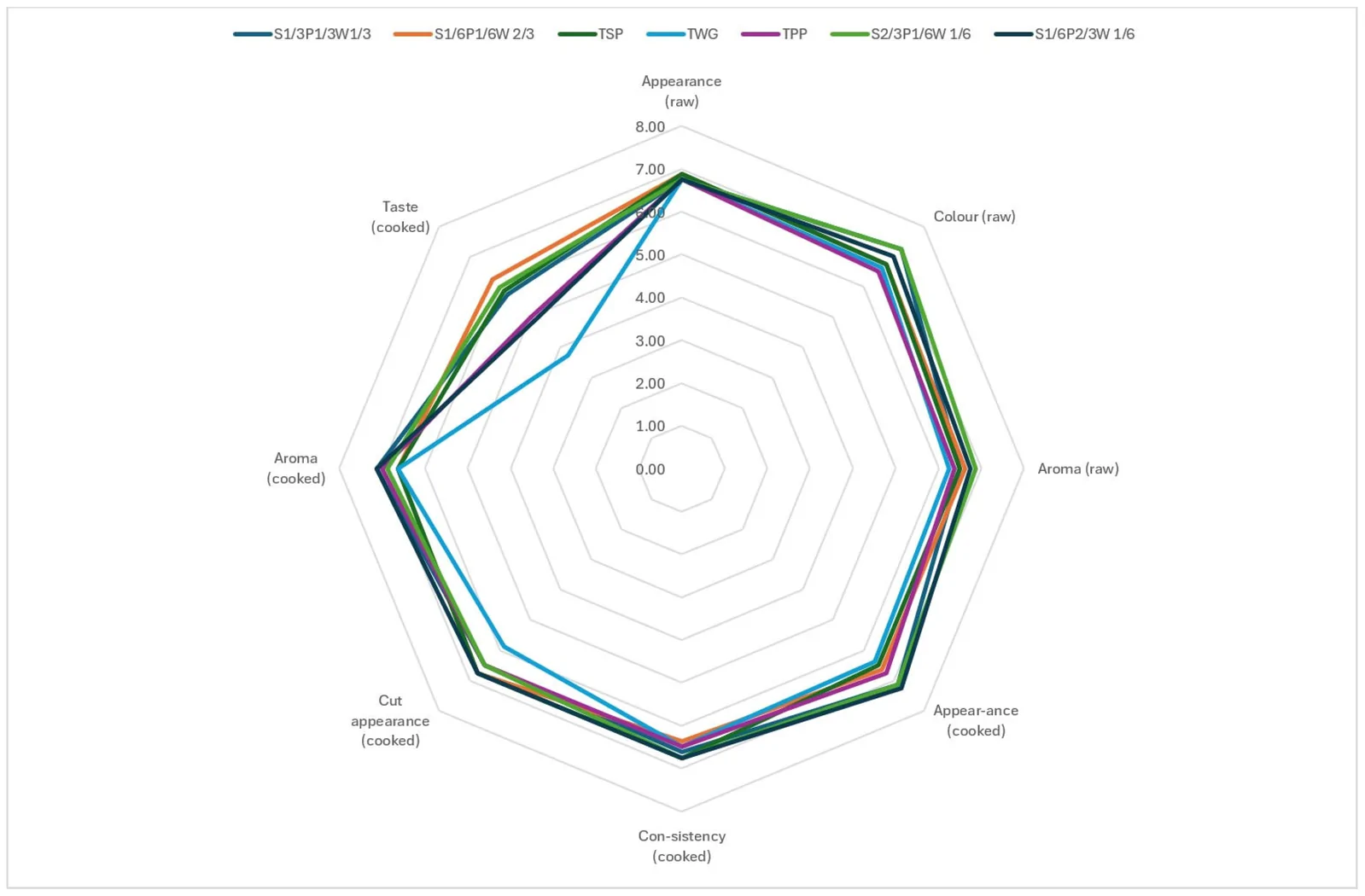
Radar plot of the sensory profiles of the model plant-based meat analogue formulations.

The scores for most sensory characteristics fell within the range from 5.88 to 7.25 points. Significant differences between the formulations were established for two of the eight characteristics—taste and appearance of the cooked samples (Figure 2). For the colour of the raw mixtures, the effect of formulation was significant according to the results of analysis of variance; however, Tukey’s test did not separate any pair. For the remaining characteristics, no differences between the formulations were revealed.

The greatest differences were noted for taste, where the range of mean scores amounted to 2.5 points. The lowest score was obtained by TWG (3.75 ± 0.16), which formed a separate group. The highest scores were obtained by S_1_/_6_P_1_/_6_W_2_/_3_ (6.25 ± 0.16) and S_2_/_3_P_1_/_6_W_1_/_6_ (6.00 ± 0.19), which did not differ significantly from each other. It is noteworthy that the S_1_/_6_P_1_/_6_W_2_/_3_ formulation contained 66.7% textured wheat gluten—that is, the same component which, as the sole protein source, gave the lowest taste score did not reduce the score when included in a blend.

For the appearance of the cooked samples, the lowest score was again obtained by TWG (6.38 ± 0.26) and the highest by S_1_/_6_P_2_/_3_W_1_/_6_ (7.25 ± 0.16); the remaining formulations occupied an intermediate position and did not differ significantly from either extreme.

The scores obtained for the eight attributes were transformed into individual desirability functions and combined into a composite sensory index (Table 9).

The desirability functions obtained for each model PBMA minced mixture make it possible to combine the original multi-criteria sensory assessments into a generalized desirability function, or composite sensory index (CSI), for use as the RESPONSE in conducting the subsequent regression analysis. The composite sensory index varied from 0.39 for TWG to 0.60 for the formulations S_2/3_P_1/6_W_1/6_ and S_1/6_P_2/3_W_1/6_; the remaining formulations had intermediate values.

### 3.7. Regression Analysis of the Model Minced Mixtures of the Plant-Based Meat Analogue

The proportions of the three types of textured plant proteins were taken as predictors, and the composite sensory index calculated from the sensory evaluation data and the hardness of the raw minced mixtures measured instrumentally were taken as responses. The results are presented in Table 10 and Table 11.

As can be seen from the results of the analysis, all three protein components exerted a positive influence on the composite sensory index. Among the factors, TSP makes the greatest contribution with the highest coefficient of 0.5528; increasing its proportion in the minced mixture will have a positive effect on its sensory properties. TWG has the smallest positive influence—0.389.

Based on the results of the regression analysis, the following regression equation was obtained:

CSI = 0.5528 (TSP) + 0.4932 (TPP) + 0.3890 (TWG) + 0.394 (TSP × TPP) + 0.221 (TSP × TWG) + 0.511 (TPP × TWG)(8)

This regression equation, describing the dependence of the sensory indicators on the mass fraction of soy, pea, and wheat proteins, will make it possible to predict or maximize the composite sensory indicator when formulating meat analogues based on blends of plant proteins.

The regression analysis of the influence of plant proteins on the hardness indicator is described by the following equation:

Y = 7.79 TSP + 4.23 TPP + 0.87 TWG − 12.2 TSP·TPP − 1.0 TSP·TWG + 1.7 TPP·TWG(9)

**Figure 3:**
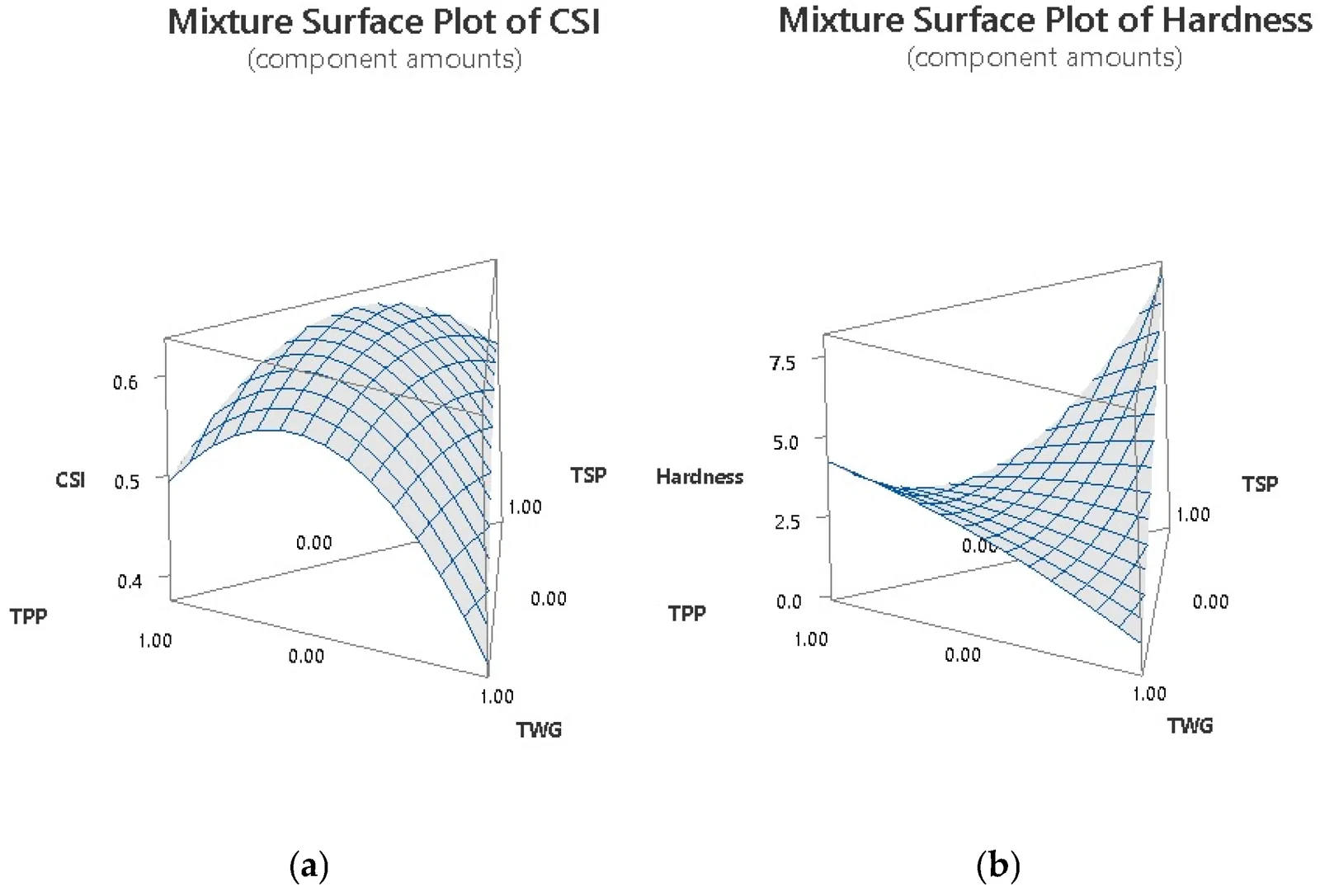
Mixture response surface plots of the plant-based meat analogues: (**a**) CSI. (**b**) Hardness.

On the basis of the regression analysis, the following response surfaces for CSI and hardness were obtained and are presented on Figure 3.

The component axes are given as proportions of the total content of textured plant proteins, taken as unity; the corresponding mass fractions are indicated in Table 2.

Figure 3 shows that the ratio of the textured protein components affects the CSI and hardness of the plant-based meat analogues. A more balanced composition of the blend promotes an increase in CSI, whereas hardness changes markedly depending on the predominance of individual protein components. This confirms the importance of optimizing the ratio of soy, pea, and wheat proteins in the development of the formulation.

**Figure 4:**
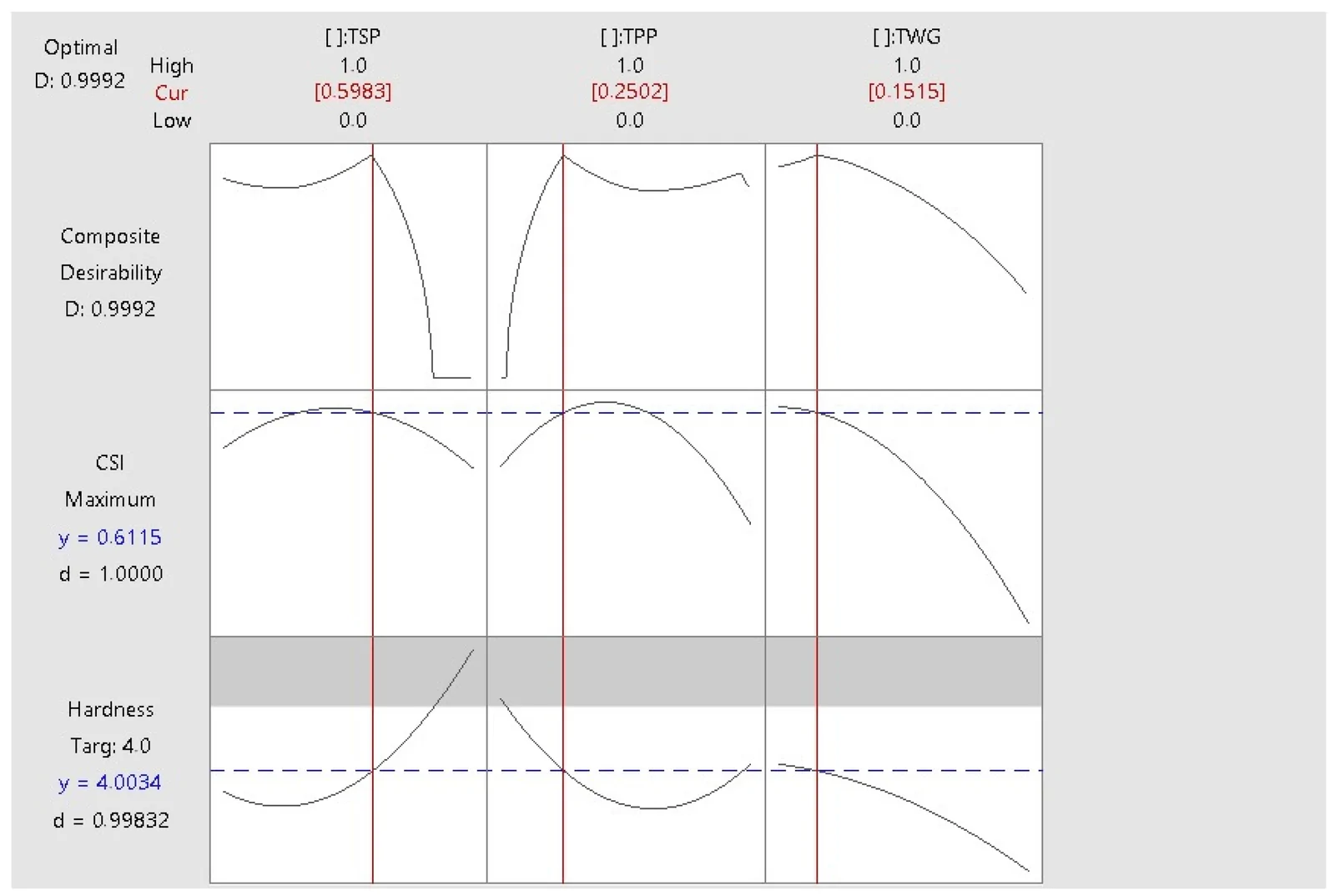
Optimization of the responses for CSI and hardness in the minced mixtures. Red vertical lines indicate the optimal proportions of the components (values in square brackets), blue dashed lines the predicted responses at these settings, and the grey area the hardness values outside the specified optimization range. D is the composite desirability and d the individual desirability of each response.

Optimization of the responses for CSI and hardness was carried out; the goal was set to maximize the CSI, while for the hardness of the raw minced mixtures, a target value of 4 N was set, corresponding to the measured hardness of the raw beef-mince control (3.83 ± 0.30 N). The optimization profile is presented in Figure 4.

As a result of optimization, a solution was obtained in the form of an optimal combination of textured vegetable which will allow for obtaining the highest possible CSI index at a product hardness comparable to that of minced meat: TSP-0.598; TPP-0.250; TWG-0.152.

## 4. Conclusions

The ratio of textured soy, pea, and wheat proteins selectively influences the properties of a comminuted plant-based meat analogue. The moisture, ash content, and caloric value of the formulations did not depend on the protein ratio, whereas the protein content varied within the range of 18.42–21.29%, and the colour, textural characteristics, and taste differed significantly.

Wheat gluten as the sole protein source formed the least strong structure (hardness of the raw mixture 0.96 N versus 7.89 N for the soy texturate) and received the lowest taste score (3.75 points). Within a blend, this effect was not manifested: the formulation with a wheat gluten proportion of 66.7% received the highest taste score (6.25 points). After heat treatment, the hardness of all formulations did not differ significantly from the beef control. The smallest colour difference from the control was shown by three formulations containing pea texturate—3.74, 5.01, and 6.74, with no significant differences between them; in terms of lightness and whiteness index, the TPP formulation did not differ from the control.

Optimization was carried out simultaneously with respect to two responses of different nature—the composite sensory index calculated from the sensory evaluation data and the instrumental hardness of the raw mixture, set equal to 4 N based on the measured value for minced beef (3.83 N). The resulting ratio of textured soy, pea, and wheat proteins was 0.598:0.250:0.152. Application of this ratio in the production of chilled and frozen comminuted semi-finished products makes it possible to obtain a product with the highest sensory characteristics at a hardness comparable to that of minced beef.

The limitations of the work include the absence of a direct sensory comparison of the developed formulations with the beef control and the interpretation of structural changes in the protein on the basis of indirect indicators without the use of methods for analysing supramolecular structure. Both directions are regarded as tasks for further research.

## Figures and Tables

**Table 1:** Recipe for plant-based patties based on extruded texturized vegetable protein (TVP).

Sample	TSP	TPP	TWG	Sunflower Oil	Water	Starch	Beet Powder	Onion Powder	Carrot Powder	Yeast Concentrate	Table Salt	Total
S_1/3_P_1/3_W_1/3_	6.72	6.72	6.72	5.6	60	8	0.4	1.84	2.4	0.8	0.8	100
S_1/6_P_1/6_W_2/3_	3.36	3.36	13.44	5.6	60	8	0.4	1.84	2.4	0.8	0.8	100
TSP	20.16	0	0	5.6	60	8	0.4	1.84	2.4	0.8	0.8	100
TWG	0	0	20.16	5.6	60	8	0.4	1.84	2.4	0.8	0.8	100
TPP	0	20.16	0	5.6	60	8	0.4	1.84	2.4	0.8	0.8	100
S_2/3_P_1/6_W_1/6_	13.44	3.36	3.36	5.6	60	8	0.4	1.84	2.4	0.8	0.8	100
S_1/6_P_2/3_W_1/6_	3.36	13.44	3.36	5.6	60	8	0.4	1.84	2.4	0.8	0.8	100

**Table 2:** Content of formulation components in the model samples of the meat analogue (mince).

Sample	Textured Soy Protein, %	Textured Pea Protein, %	Textured Wheat Gluten, %
S_1/3_P_1/3_W_1/3_	33.3	33.3	33.3
S_1/6_P_1/6_W_2/3_	16.7	16.7	66.7
TSP	100.0	0.0	0.0
TWG	0.0	0.0	100.0
TPP	0.0	100.0	0.0
S_2/3_P_1/6_W_1/6_	66.7	16.7	16.7
S_1/6_P_2/3_W_1/6_	16.7	66.7	16.7

TSP—textured soy protein; TWG—textured wheat gluten; TPP—textured pea protein.

**Table 3:** Proximate composition of protein isolates.

Sample	Moisture, %	Protein, %	Fat, %	Carbohydrates, %	Ash, %	Protein Solubility, %
Pea protein isolate	3.43 ± 0.47 ^a^	79.36 ± 0.24 ^a^	2.45 ± 0.10 ^a^	12.34 ± 0.14 ^a^	2.42 ± 0.05 ^a^	16.21 ± 1.39 ^a^
Soy protein isolate	7.8 ± 0.16 ^b^	78.36 ± 0.24 ^a^	0.12 ± 0.01 ^b^	9.93 ± 0.12 ^b^	3.81 ± 0.02 ^b^	7.07 ± 0.40 ^b^
Wheat protein isolate	3.6 ± 0.08 ^a^	69.37 ± 0.60 ^b^	3.33 ± 0.23 ^c^	20.05 ± 0.17 ^c^	3.65 ± 0.11 ^b^	11.65 ± 0.26 ^c^

Results are presented as mean ± standard error of the mean (*n* = 3). Different superscript letters within a column indicate statistically significant differences between the isolates (*p* < 0.05). Protein solubility was determined at pH 7.0.

**Table 4:** Physiochemical composition and pH of the model PBMA formulations and minced beef (control).

Sample	pH	Moisture, %	Protein, %	Fat, %	Carbohydrates, %	Ash, %	Caloric Value, kcal/100 g
S_1/3_P_1/3_W_1/3_	7.12 ± 0.02 ^a^	60.81 ± 0.61	19.86 ± 0.03 ^bc^	6.20 ± 0.21 ^b^	11.08 ± 0.9 ^ab^	2.05 ± 0.07	182.15 ± 1.64 ^b^
S_1/6_P_1/6_W_2/3_	7.09 ± 0.03 ^a^	60.56 ± 0.66	18.78 ± 0.26 ^de^	6.18 ± 0.06 ^b^	12.34 ± 0.88 ^ab^	2.14 ± 0.18	182.24 ± 2.63 ^b^
TSP	6.65 ± 0.31 ^b^	61.09 ± 0.44	21.29 ± 0.27 ^a^	6.04 ± 0.44 ^b^	9.60 ± 0.83 ^b^	1.99 ± 0.26	181.11 ± 1.72 ^b^
TWG	7.06 ± 0.06 ^a^	59.87 ± 0.61	18.42 ± 0.18 ^e^	5.99 ± 0.52 ^b^	13.61 ± 0.81 ^a^	2.11 ± 0.14	183.97 ± 2.68 ^b^
TPP	7.05 ± 0.04 ^a^	60.11 ± 0.27	18.72 ± 0.1 ^de^	6.06 ± 0.38 ^b^	13.13 ± 0.29 ^a^	1.97 ± 0.08	184.02 ± 2.83 ^b^
S_2/3_P_1/6_W_1/6_	7.02 ± 0.02 ^a^	60.54 ± 0.71	20.43 ± 0.08 ^b^	6.08 ± 0.11 ^b^	10.98 ± 0.71 ^ab^	1.96 ± 0.06	183.18 ± 3.23 ^b^
S_1/6_P_2/3_W_1/6_	7.11 ± 0.09 ^a^	60.92 ± 0.33	19.40 ± 0.07 ^cd^	6.09 ± 0.2 ^b^	11.62 ± 0.06 ^ab^	1.97 ± 0.05	181.32 ± 2.13 ^b^
Control	6.23 ± 0.04 ^c^	62.15 ± 0.56	18.55 ± 0.05 ^e^	16.63 ± 0.27 ^a^	1.01 ± 0.45 ^c^	1.66 ± 0.14	229.28 ± 3.03 ^a^

Results are expressed as mean ± standard error of the mean (*n* = 3). Different letters indicate significant differences in the same column (*p* < 0.05). Moisture and ash contents did not differ significantly between the samples (*p* > 0.05); superscript letters are therefore not given for these two parameters.

**Table 5:** Colour characteristics of the surface of the plant-based meat analogues after heat treatment.

Sample	After Thermal Treatment			ΔE*	Whiteness (WI)
	L*	a*	b*		
S_1/3_P_1/3_W_1/3_	46.35 ± 0.24 ^a^	7.36 ± 0.42 ^c^	10.70 ± 0.88 ^b^	12.04 ± 0.79 ^a^	44.78 ± 0.39 ^a^
S_1/6_P_1/6_W_2/3_	44.65 ± 0.57 ^ab^	6.66± 1.06 ^c^	20.49 ± 0.99 ^a^	14.30 ± 1.05 ^a^	40.58 ± 0.76 ^b^
TSP	42.17 ± 0.54 ^c^	9.8± 0.51 ^abc^	21.22 ± 1.00 ^a^	12.98 ± 0.82 ^a^	37.61 ± 0.67 ^c^
TWG	42.54 ± 0.11 ^bc^	8.93 ± 0.96 ^bc^	9.61 ± 1.09 ^b^	8.56 ± 0.49 ^b^	41.02 ± 0.14 ^b^
TPP	36.38 ± 0.78 ^ef^	10.23 ± 0.64 ^abc^	12.70 ± 0.80 ^b^	3.74 ± 0.45 ^c^	34.31 ± 0.93 ^de^
S_2/3_P_1/6_W_1/6_	38.46 ± 0.31 ^de^	11.45 ± 0.65 ^ab^	10.26 ± 0.82 ^b^	5.01 ± 0.51 ^c^	36.56 ± 0.36 ^cd^
S_1/6_P_2/3_W_1/6_	39.27 ± 0.39 ^d^	12.74 ± 1.13 ^a^	9.83 ± 0.79 ^b^	6.74 ± 0.55 ^bc^	37.16 ± 0.66 ^cd^
Control	34.46 ± 0.50 ^f^	8.76 ± 0.30 ^bc^	11.03 ± 0.63 ^b^	-	32.95 ± 0.46 ^e^

Values denoted by different superscripts within the column are significantly different (*p* ≤ 0.05). L* = Lightness; +a* = Redness, +b* = Yellowness. The control sample served as the reference basis for calculating ΔE*; therefore, no value is given for it.

**Table 6:** Techno-functional properties of the plant-based meat analogues in comparison with minced meat (control).

Sample	WAI, g/g	WAC, g/g	OAC, g/g
S_1/3_P_1/3_W_1/3_	2.35 ± 0.47	1.19 ± 0.1 ^ab^	1.92 ± 0.1 ^a^
S_1/6_P_1/6_W_2/3_	2.25 ± 0.5	1.18 ± 0.06 ^ab^	1.76 ± 0.15 ^a^
TSP	2.53 ± 0.42	1.27 ± 0.02 ^a^	1.98 ± 0.09 ^a^
TWG	2.33 ± 0.5	1.16 ± 0.05 ^ab^	1.79 ± 0.05 ^a^
TPP	2.16 ± 0.61	0.9 ± 0.07 ^b^	2.08 ± 0.26 ^a^
S _2/3_P_1/6_W_1/6_	1.98 ± 0.62	0.95 ± 0.1 ^ab^	1.82 ± 0.15 ^a^
S_1/6_P_2/3_W_1/6_	1.54 ± 0.19	1.04 ± 0.09 ^ab^	2.03 ± 0.2 ^a^
Control	0.890 ± 0.04	0.9 ± 0.03 ^b^	0.98 ± 0.06 ^b^

Results are expressed as mean ± standard error of the mean (*n* = 3). Different letters indicate significant differences in the same column (*p* < 0.05). WAI values did not differ significantly between the samples (*p* > 0.05); superscript letters are therefore not given for this parameter.

**Table 7:** Texture profile analysis of the minced mixtures before and after cooking.

Sample	Hardness, N	Cohesiveness	Springiness	Gumminess, N	Chewiness, N·mm	Adhesiveness, N	Elasticity, %
Raw minced meat							
S_1/3_P_1/3_W_1/3_	3.84 ± 0.36 ^bc^	0.80 ± 0.002 ^a^	0.78 ± 0.01 ^bc^	3.09 ± 0.3 ^bc^	2.41 ± 0.25 ^b^	−0.4 ± 0.07 ^bcd^	9.95 ± 0.45 ^c^
S_1/6_P_1/6_W_2/3_	1.96 ± 0.24 ^de^	0.34 ± 0.003 ^c^	0.46 ± 0.005 ^d^	0.66 ± 0.08 ^d^	0.3 ± 0.04 ^d^	−0.2 ± 0.09 ^ab^	5.57 ± 0.52 ^de^
TSP	7.89 ± 0.67 ^a^	0.83 ± 0.001 ^a^	0.81 ± 0.004 ^b^	6.57 ± 0.55 ^a^	5.34 ± 0.44 ^a^	−0.04 ± 0.02 ^a^	36.37 ± 1.24 ^a^
TWG	0.96 ± 0.05 ^e^	0.38 ± 0.002 ^c^	0.81 ± 0.004 ^b^	0.37 ± 0.02 ^d^	0.3 ± 0.01 ^d^	−0.25 ± 0.02 ^abc^	1.75 ± 0.38 ^e^
TPP	4.32 ± 0.22 ^b^	0.83 ± 0.007 ^a^	0.77 ± 0.014 ^bc^	3.59 ± 0.15 ^b^	2.77 ± 0.08 ^b^	−0.51 ± 0.03 ^d^	6.13 ± 0.26 ^cd^
S_2/3_P_1/6_W_1/6_	4.27 ± 0.28 ^bc^	0.82 ± 0.01 ^a^	0.74 ± 0.019 ^c^	3.49 ± 0.27 ^bc^	2.59 ± 0.26 ^b^	−0.45 ± 0.03 ^cd^	17.39 ± 1.06 ^b^
S_1/6_P_2/3_W_1/6_	2.71 ± 0.02 ^cd^	0.82 ± 0.047 ^a^	0.9 ± 0.021 ^a^	2.22 ± 0.14 ^c^	2.02 ± 0.17 ^bc^	−0.49 ± 0.02 ^d^	3.67 ± 0.15 ^de^
Control	3.83 ± 0.3 ^bc^	0.6 ± 0.003 ^b^	0.5 ± 0.006 ^d^	2.3 ± 0.19 ^c^	1.14 ± 0.11 ^cd^	−0.53 ± 0.001 ^d^	15.29 ± 1.53 ^b^
Minced meat as fried patties							
S_1/3_P_1/3_W_1/3_	34.76 ± 4.23 ^a^	0.92 ± 0.004 ^a^	0.86 ± 0.01	32.03 ± 3.79 ^a^	27.63 ± 3.11 ^a^	–	70.75 ± 1.02 ^ab^
S_1/6_P_1/6_W_2/3_	38.07 ± 4.51 ^a^	0.92 ± 0.003 ^a^	0.89 ± 0.01	34.92 ± 4.22 ^a^	31.2 ± 3.73 ^a^	–	71.43 ± 2.39 ^a^
TSP	18.40 ± 2.19 ^b^	0.93 ± 0.004 ^a^	0.92 ± 0.01	17.06 ± 2.06 ^bc^	15.75 ± 1.99 ^bc^	–	48.37 ± 6.74 ^bcd^
TWG	28.4 ± 3.73 ^ab^	0.93 ± 0.001 ^a^	0.86 ± 0.01	26.51 ± 3.51 ^ab^	22.78 ± 3.14 ^ab^	–	73.51 ± 3.49 ^a^
TPP	14.07 ± 0.63 ^b^	0.86 ± 0.003 ^b^	0.8 ± 0.005	12.06 ± 0.56 ^c^	9.69 ± 0.4 ^c^	–	38.78 ± 9.83 ^cde^
S_2/3_P_1/6_W_1/6_	18.08 ± 1.77 ^b^	0.84 ± 0.004 ^b^	0.79 ± 0.01	15.15 ± 1.47 ^bc^	11.93 ± 1.21 ^bc^	–	54.8 ± 2.08 ^abc^
S_1/6_P_2/3_W_1/6_	17.67 ± 1.41 ^b^	0.87 ± 0.011 ^b^	0.8 ± 0.02	15.27 ± 1.04 ^bc^	12.12 ± 0.55 ^bc^	–	22.39 ± 2.34 ^e^
Control	23.69 ± 2.68 ^ab^	0.77 ± 0.024 ^c^	0.85 ± 0.08	18.45 ± 2.7 ^bc^	15.3 ± 0.6 ^bc^	−0.08 ± 0.03	31.39 ± 0.89 ^de^

Results are expressed as mean ± standard error of the mean (*n* = 3). Different letters in the same column indicate significant differences (*p* < 0.05). Control—minced meat. Springiness values of the cooked samples did not differ significantly (*p* > 0.05); superscript letters are therefore not given for this parameter. Adhesiveness of the cooked plant-based samples was below the detection limit and is not reported. Control—minced meat.

**Table 8:** Sensory evaluation scores of the model plant-based meat analogue formulations.

Sample	Appearance (Raw)	Colour (Raw)	Aroma (Raw)	Appearance (Cooked)	Consistency (Cooked)	Cut Appearance (Cooked)	Aroma (Cooked)	Taste (Cooked)
S _1/3_P_1/3_W_1/3_	6.75 ± 0.16	7.25 ± 0.16	6.5 ± 0.27	7.13 ± 0.13 ^ab^	6.63 ± 0.18	6.50 ± 0.27	7.13 ± 0.13	5.75 ± 0.16 ^abc^
S_1/6_P_1/6_W_2/3_	6.88 ± 0.13	6.75 ± 0.16	6.63 ± 0.18	6.63 ± 0.18 ^ab^	6.38 ± 0.18	6.75 ± 0.16	6.63 ± 0.18	6.25 ± 0.16 ^a^
TSP	6.88 ± 0.13	6.75 ± 0.16	6.50 ± 0.19	6.50 ± 0.27 ^ab^	6.75 ± 0.16	6.75 ± 0.16	6.63 ± 0.18	5.88 ± 0.23 ^ab^
TWG	6.75 ± 0.16	6.63 ± 0.18	6.25 ± 0.25	6.38 ± 0.26 ^b^	6.50 ± 0.19	5.88 ± 0.3	6.63 ± 0.18	3.75 ± 0.16 ^d^
TPP	6.75 ± 0.16	6.50 ± 0.19	6.38 ± 0.26	6.75 ± 0.16 ^ab^	6.50 ± 0.19	6.50 ± 0.19	7.00 ± 0.19	5.00 ± 0.27 ^bc^
S_2/3_P_1/6_W_1/6_	6.75 ± 0.16	7.25 ± 0.16	6.88 ± 0.13	7.13 ± 0.13 ^ab^	6.75 ± 0.16	6.50 ± 0.27	6.88 ± 0.23	6.00 ± 0.19 ^a^
S_1/6_P_2/3_W_1/6_	6.75 ± 0.16	7.00 ± 0.27	6.75 ± 0.16	7.25 ± 0.16 ^a^	6.75 ± 0.16	6.75 ± 0.16	7.13 ± 0.23	4.88 ± 0.23 ^c^

Results are expressed as mean ± standard error of the mean (*n* = 8). Different letters within a column indicate significant differences between samples (one-way ANOVA followed by Tukey’s HSD test, *p* < 0.05). Columns without letters showed no significant pairwise differences.

**Table 9:** Partial and generalized desirability functions for the composite sensory indicator, calculated according to Equations (5)–(7).

Model		Appearance (Raw)	Colour (Raw)	Aroma (Raw)	Appearance (Cooked)	Consistency (Cooked)	Cut Appearance (Cooked)	Aroma (Cooked)	Taste (Cooked)	CSI (*D*)
*d*1	S_1/3_P_1/3_W_1/3_	0.368	0.692	0.512	0.654	0.598	0.613	0.692	0.638	0.59
*d*2	S_1/6_P_1/6_W_2/3_	0.692	0.488	0.578	0.472	0.368	0.692	0.368	0.692	0.53
*d*3	TSP	0.692	0.488	0.512	0.420	0.692	0.692	0.368	0.652	0.55
*d*4	TWG	0.368	0.429	0.368	0.368	0.488	0.368	0.368	0.368	0.39
*d*5	TPP	0.368	0.368	0.441	0.521	0.488	0.613	0.624	0.545	0.49
*d*6	S_2/3_P_1/6_W_1/6_	0.368	0.692	0.692	0.654	0.692	0.613	0.545	0.666	0.60
*d*7	S_1/6_P_2/3_W_1/6_	0.368	0.598	0.638	0.692	0.692	0.692	0.692	0.529	0.60

The individual desirability functions for each sensory attribute and the overall desirability function *D*, adopted as the composite sensory index (CSI), are presented. The values are dimensionless and range from 0 to 1. The designations *d*1–*d*7 correspond to the model formulations.

**Table 10:** Regression coefficients of the composite sensory index.

Term	Coef	SE Coef	*t*-Value	*p*-Value	VIF	R-sq, %
TSP	0.5528	0.0114	*	*	1.60	99.63
TWG	0.3890	0.0114	*	*	1.60	99.63
TPP	0.4932	0.0114	*	*	1.60	99.63
TSP×TPP	0.394	0.129	3.05	0.202	4.83	99.63
TSP×TWG	0.221	0.129	1.71	0.337	4.83	99.63
TPP×TWG	0.511	0.129	3.96	0.157	4.83	99.63

The variance inflation factor was 1.60 for the linear terms and 4.83 for the pairwise interactions. For the linear terms of the mixture model without an intercept, *t*- and *p*-values are not calculated and are marked as asterisk (*).

**Table 11:** Regression coefficients of the hardness indicator.

Term	Coef	SE Coef	*t*-Value	*p*-Value	VIF	R-sq, %
TSP	7.79	1.04	*	*	1.60	99.35
TPP	4.23	1.04	*	*	1.60	99.35
TWG	0.87	1.04	*	*	1.60	99.35
TSP×TPP	−12.2	11.8	−1.03	0.489	4.83	99.35
TSP×TWG	−1.0	11.8	−0.08	0.947	4.83	99.35
TPP×TWG	1.7	11.8	0.14	0.910	4.83	99.35

The variance inflation factor was 1.60 for the linear terms and 4.83 for the pairwise interactions. For the linear terms of the mixture model without an intercept, *t*- and *p*-values are not calculated and are marked as asterisk (*).

## Data Availability

The original contributions presented in this study are included in the article. Further inquiries can be directed to the corresponding author.

## Abbreviations

The following abbreviations are used in this manuscript:

PBMA	Plant-based meat analogue
WAC	Water absorption capacity
WAI	Water absorption index
OAC	Oil absorption capacity
CSI	Composite sensory index
TPP	Textured pea protein
TSP	Textured soy protein
TWG	Textured wheat gluten

## References

[1] Bureau of National Statistics of the Agency for Strategic Planning and Reforms of the Republic of Kazakhstan Consumption of Food Products in Households of the Republic of Kazakhstan. https://stat.gov.kz/ru/industries/labor-and-income/stat-life/publications/365313/.

[2] Septiani S., Kusumastuti R.D., Gayatri G. (2026). Mapping the digital landscape of organic food: A bibliometric and systematic review with future directions. Br. Food J..

[3] Draper J., Revilla M., Titchenal A., Caacbay N., Calabrese A., Chun C., Gibby C., Hara S., Langfelder G., Yang Y. (2020). Proteins, diet, and personal choices. Human Nutrition.

[4] Webb D., Dogan H., Li Y., Alavi S. (2023). Physico-chemical properties and texturization of pea, wheat and soy proteins using extrusion and their application in plant-based meat. Foods.

[5] Narale B.A., Mounika A., Shanmugam A. (2024). Modifications of physicochemical, functional, structural, and nutritional properties of a field bean protein isolate obtained using batch and continuous ultrasound systems. Sustain. Food Technol..

[6] Sun C., Ge J., He J., Gan R., Fang Y. (2021). Processing, quality, safety, and acceptance of meat analogue products. Engineering.

[7] Wang J., Kadyan S., Ukhanov V., Cheng J., Nagpal R., Cui L. (2022). Recent advances in the health benefits of pea protein (*Pisum sativum*): Bioactive peptides and the interaction with the gut microbiome. Curr. Opin. Food Sci..

[8] Richard C., Jacquenet S., Sergeant P., Moneret-Vautrin D. (2015). Cross-reactivity of a new food ingredient, dun pea, with legumes, and risk of anaphylaxis in legume allergic children. Eur. Ann. Allergy Clin. Immunol..

[9] Rao V., Poonia A. (2023). Protein characteristics, amino acid profile, health benefits and methods of extraction and isolation of proteins from some pseudocereals—A review. Food Prod. Process. Nutr..

[10] García Arteaga V., Kraus S., Schott M., Muranyi I., Schweiggert-Weisz U., Eisner P. (2021). Screening of twelve pea (*Pisum sativum* L.) cultivars and their isolates focusing on the protein characterization, functionality, and sensory profiles. Foods.

[11] Abrams E.M., Gerstner T.V. (2015). Allergy to cooked, but not raw, peas: A case series and review. Allergy Asthma Clin. Immunol..

[12] Shewry P.R., Halford N.G. (2002). Cereal seed storage proteins: Structures, properties and role in grain utilization. J. Exp. Bot..

[13] Dekkers B.L., Boom R.M., van der Goot A.J. (2018). Structuring processes for meat analogues. Trends Food Sci. Technol..

[14] Day L. (2011). Wheat gluten: Production, properties and application. Handbook of Food Proteins.

[15] Wieser H. (2007). Chemistry of gluten proteins. Food Microbiol..

[16] Malav O., Talukder S., Gokulakrishnan P., Chand S. (2015). Meat analog: A review. Crit. Rev. Food Sci. Nutr..

[17] Schreuders F.K., Dekkers B.L., Bodnár I., Erni P., Boom R.M., van der Goot A.J. (2019). Comparing structuring potential of pea and soy protein with gluten for meat analogue preparation. J. Food Eng..

[18] Lee J.-S., Kim S., Jeong Y.J., Choi I., Han J. (2023). Impact of interactions between soy and pea proteins on quality characteristics of high-moisture meat analogues prepared via extrusion cooking process. Food Hydrocoll..

[19] Flory J., Xiao R., Li Y., Dogan H., Talavera M.J., Alavi S. (2023). Understanding protein functionality and its impact on quality of plant-based meat analogues. Foods.

[20] Webb D., Li Y., Alavi S. (2023). Chemical and physicochemical features of common plant proteins and their extrudates for use in plant-based meat. Trends Food Sci. Technol..

[21] Elshamy A.F., Rosentrater K.A., Ghnimi S., Hossain M.K., Lammers V., Heinz V., Diakité M. (2025). High-moisture meat analogs produced from dry-fractionated Faba bean, yellow pea, and functional soy proteins: Effects of mixture design and extrusion parameters on texture properties. Innov. Food Sci. Emerg. Technol..

[22] Chitra Devi V., Devanampriyan R., Kayethri D., Sankari R., Premalatha J., Sathish Raam R., Mothil S. (2025). Optimization and process validation of freeze-structured meat substitute using machine learning models. J. Food Process Eng..

[23] Song H.Y., Jeong D.H., Jung Y.J., Jo Y.J., Lee J., Jeong H.S. (2026). Optimization of mixing ratio for a TVP-based alternative sausage using mixture experimental design. Food Sci. Biotechnol..

[24] Madhav C.D., Bhalerao P.P., Dabade A., Hundare S., Bhushette P., Deshmukh S.D., Sonawane S.K. (2025). Development and characterization of jackfruit-based vegan patties: A D-optimal mixture design approach. J. Food Meas. Charact..

[25] Karam M.C., Petit J., Zimmer D., Djantou E.B., Scher J. (2016). Effects of drying and grinding in production of fruit and vegetable powders: A review. J. Food Eng..

[26] Srikanlaya C., Therdthai N. (2024). Characterization of plant-based meat treated with hot air and microwave heating. Foods.

[27] King D.A., Hunt M.C., Barbut S., Claus J.R., Cornforth D.P., Joseph P., Kim Y.H.B., Lindahl G., Mancini R.A., Nair M.N. (2023). American Meat Science Association guidelines for meat color measurement. Meat Muscle Biol..

[28] Ketnawa S., Chaijan M., Grossmann L., Rawdkuen S. (2024). High-moisture soy protein-mushroom-based meat analogue: Physicochemical, structural properties and its application. Int. J. Food Sci. Technol..

[29] De Angelis D., Kaleda A., Pasqualone A., Vaikma H., Tamm M., Tammik M.-L., Squeo G., Summo C. (2020). Physicochemical and sensorial evaluation of meat analogues produced from dry-fractionated pea and oat proteins. Foods.

[30] Dunne R.A., Darwin E.C., Medina V.A.P., Levenston M.E., Pierre S.R.S., Kuhl E. (2025). Texture profile analysis and rheology of plant-based and animal meat. Food Res. Int..

[31] Mustafa R.R., Amin N.S., Abdullah Q., Mohd Hanafiah N.S., Mohd Bakri N.F., Yusoff I.M. (2026). A Comparative Study of Nutritional, Microbiological and Sensory Profiles in Plant-Based Meat Analogues and Real Meat Patty. Food Bioprocess Technol..

[32] Cutroneo S., Angelino D., Tedeschi T., Pellegrini N., Martini D., Group S.Y.W. (2022). Nutritional quality of meat analogues: Results from the food labelling of Italian products (FLIP) project. Front. Nutr..

[33] Bakhsh A., Lee S.-J., Lee E.-Y., Hwang Y.-H., Joo S.-T. (2021). Evaluation of rheological and sensory characteristics of plant-based meat analog with comparison to beef and pork. Food Sci. Anim. Resour..

[34] Xie Y., Cai L., Zhou G., Li C. (2024). Comparison of nutritional profile between plant-based meat analogues and real meat: A review focusing on ingredients, nutrient contents, bioavailability, and health impacts. Food Res. Int..

[35] Berrazaga I., Micard V., Gueugneau M., Walrand S. (2019). The role of the anabolic properties of plant-versus animal-based protein sources in supporting muscle mass maintenance: A critical review. Nutrients.

[36] Samard S., Ryu G.H. (2019). A comparison of physicochemical characteristics, texture, and structure of meat analogue and meats. J. Sci. Food Agric..

[37] Chiang J.H., Loveday S.M., Hardacre A.K., Parker M.E. (2019). Effects of soy protein to wheat gluten ratio on the physicochemical properties of extruded meat analogues. Food Struct..

[38] Xiao Z., Jiang R., Huo J., Wang H., Li H., Su S., Gao Y., Duan Y. (2022). Rice bran meat analogs: Relationship between extrusion parameters, apparent properties and secondary structures. LWT.

[39] Siddiqui S.A., Khalifa I., Yin T., Morsy M.K., Khoder R.M., Salauddin M., Farzana W., Sharma S., Khalid N. (2024). Valorization of plant proteins for meat analogues design—A comprehensive review. Eur. Food Res. Technol..

[40] Zhang R., Yang Y., Liu Q., Xu L., Bao H., Ren X., Jin Z., Jiao A. (2023). Effect of wheat gluten and peanut protein ratio on the moisture distribution and textural quality of high-moisture extruded meat analogs from an extruder response perspective. Foods.

[41] Flory J., Alavi S. (2024). Use of hydration properties of proteins to understand their functionality and tailor texture of extruded plant-based meat analogues. J. Food Sci..

[42] Batista A.P., Portugal C.A., Sousa I., Crespo J.G., Raymundo A. (2005). Accessing gelling ability of vegetable proteins using rheological and fluorescence techniques. Int. J. Biol. Macromol..

[43] Wang L., Yu J., Xia S., Tu X., Yang L., Xue Y., Xue C. (2024). Alaska pollock surimi-based meat analogs by high-moisture extrusion: Effect of konjac glucomannan/curdlan/carrageenan/sodium alginate on fibrous structure formation. Food Chem..

[44] Jiang L., Zhang H., Zhang J., Liu S., Tian Y., Cheng T., Guo Z., Wang Z. (2024). Improve the fiber structure and texture properties of plant-based meat analogues by adjusting the ratio of soy protein isolate (SPI) to wheat gluten (WG). Food Chem. X.

[45] Wang K.-Q., Luo S.-Z., Zhong X.-Y., Cai J., Jiang S.-T., Zheng Z. (2017). Changes in chemical interactions and protein conformation during heat-induced wheat gluten gel formation. Food Chem..

[46] Vu G., Zhou H., McClements D.J. (2022). Impact of cooking method on properties of beef and plant-based burgers: Appearance, texture, thermal properties, and shrinkage. J. Agric. Food Res..

